# Bibliometric analysis of artificial intelligence for biotechnology and applied microbiology: Exploring research hotspots and frontiers

**DOI:** 10.3389/fbioe.2022.998298

**Published:** 2022-10-07

**Authors:** Dongyu Xu, Bing Liu, Jian Wang, Zhichang Zhang

**Affiliations:** ^1^ Department of Computer, School of Intelligent Medicine, China Medical University, Shenyang, Liaoning, China; ^2^ Department of Bone Oncology, The People’s Hospital of Liaoning Province, Shenyang, Liaoning, China; ^3^ Department of Pathogenic Biology, School of Basic Medicine, China Medical University, Shenyang, Liaoning, China

**Keywords:** artificial intelligence, biotechnology, bibliometric, deep learning, machine learning, applied microbiology

## Abstract

**Background:** In the biotechnology and applied microbiology sectors, artificial intelligence (AI) has been extensively used in disease diagnostics, drug research and development, functional genomics, biomarker recognition, and medical imaging diagnostics. In our study, from 2000 to 2021, science publications focusing on AI in biotechnology were reviewed, and quantitative, qualitative, and modeling analyses were performed.

**Methods:** On 6 May 2022, the Web of Science Core Collection (WoSCC) was screened for AI applications in biotechnology and applied microbiology; 3,529 studies were identified between 2000 and 2022, and analyzed. The following information was collected: publication, country or region, references, knowledgebase, institution, keywords, journal name, and research hotspots, and examined using VOSviewer and CiteSpace V bibliometric platforms.

**Results:** We showed that 128 countries published articles related to AI in biotechnology and applied microbiology; the United States had the most publications. In addition, 584 global institutions contributed to publications, with the Chinese Academy of Science publishing the most. Reference clusters from studies were categorized into ten headings: deep learning, prediction, support vector machines (SVM), object detection, feature representation, synthetic biology, amyloid, human microRNA precursors, systems biology, and single cell RNA-Sequencing. Research frontier keywords were represented by microRNA (2012–2020) and protein-protein interactions (PPIs) (2012–2020).

**Conclusion:** We systematically, objectively, and comprehensively analyzed AI-related biotechnology and applied microbiology literature, and additionally, identified current hot spots and future trends in this area. Our review provides researchers with a comprehensive overview of the dynamic evolution of AI in biotechnology and applied microbiology and identifies future key research areas.

## Introduction

Since the beginning of the 21st century, the life sciences and biotechnology and applied microbiology sectors have exemplified mankind’s technological and revolutionary evolution. In these sectors, among the top 10 scientific breakthroughs published by science journals in recent decades, more than half of research outputs were revolutionarily innovative and breakthrough in nature. Emerging biological sectors include, biomedicine, bio-based chemicals, bioenergy, and genetically modified crop technology (Celikkanat Ozan and Baran, 2013). These areas are cutting-edge, and next-generation biotechnology industries are anticipated to develop rapidly in the future (Lee, 2016). As the front end of these biological industries and value chains, biotechnology and applied microbiology research has adopted a leading position in these industries. Therefore, exploring rapid developments and hot trends in basic biotechnology and applied microbiology research is pivotal in guiding biotechnology current achievements and developing new, downstream bio-industry markets.

AI represents advanced computer technology, and is a highly complex system integrating mathematics, statistics, probability, logic, ethics, and other disciplines. It primarily includes deep learning, machine learning, convolution and recurrent neural networks (CNN and RNN, respectively), full revolutionary networks (FCNs), and other specific methods. AI is extensively used in different industries, in particular biotechnology and the life sciences. In recent years, several major research developments have been achieved, including the AI-mediated prediction of protein structure, which was breakthrough of the year in 2021 (Baek et al., 2021). By exploiting complex simulation algorithms, AI has revolutionized disease diagnostics, drug research and development, functional genomics, biomarker recognition, and medical imaging diagnostics, and critically, has provided a vital reference point for disease diagnostic, prediction, and treatment strategies (Dlamini et al., 2020).

To facilitate AI research and progress in biotechnology and applied microbiology, bibliometric analyses and reviews are used to equip scientists with in-depth understandings of the application, its ongoing evolution, and future prospects. From a database search spanning 1 January 2000 to 31 December 2021, we used bibliometric methods to analyze scientific papers on AI applications in biotechnology and applied microbiology, including papers published in different jurisdictions and by institutions. We examined journals where AI biotechnological research studies were published, investigated the “top 10 cited studies”, and enumerated how many times popular studies were cited. We clustered the reference network of cited studies, and investigated the subject knowledge base. Research hotspots were identified using burst keywords, which provided invaluable indicators for future research. Our research remit was to provide researchers with a macro understanding and micro analysis of the AI biotechnological field. When compared with traditional systematic reviews, we provided an intuitive, timely, and logical framework to track biotechnological developments and explore specific knowledge areas.

## Methods

**FIGURE 1:**
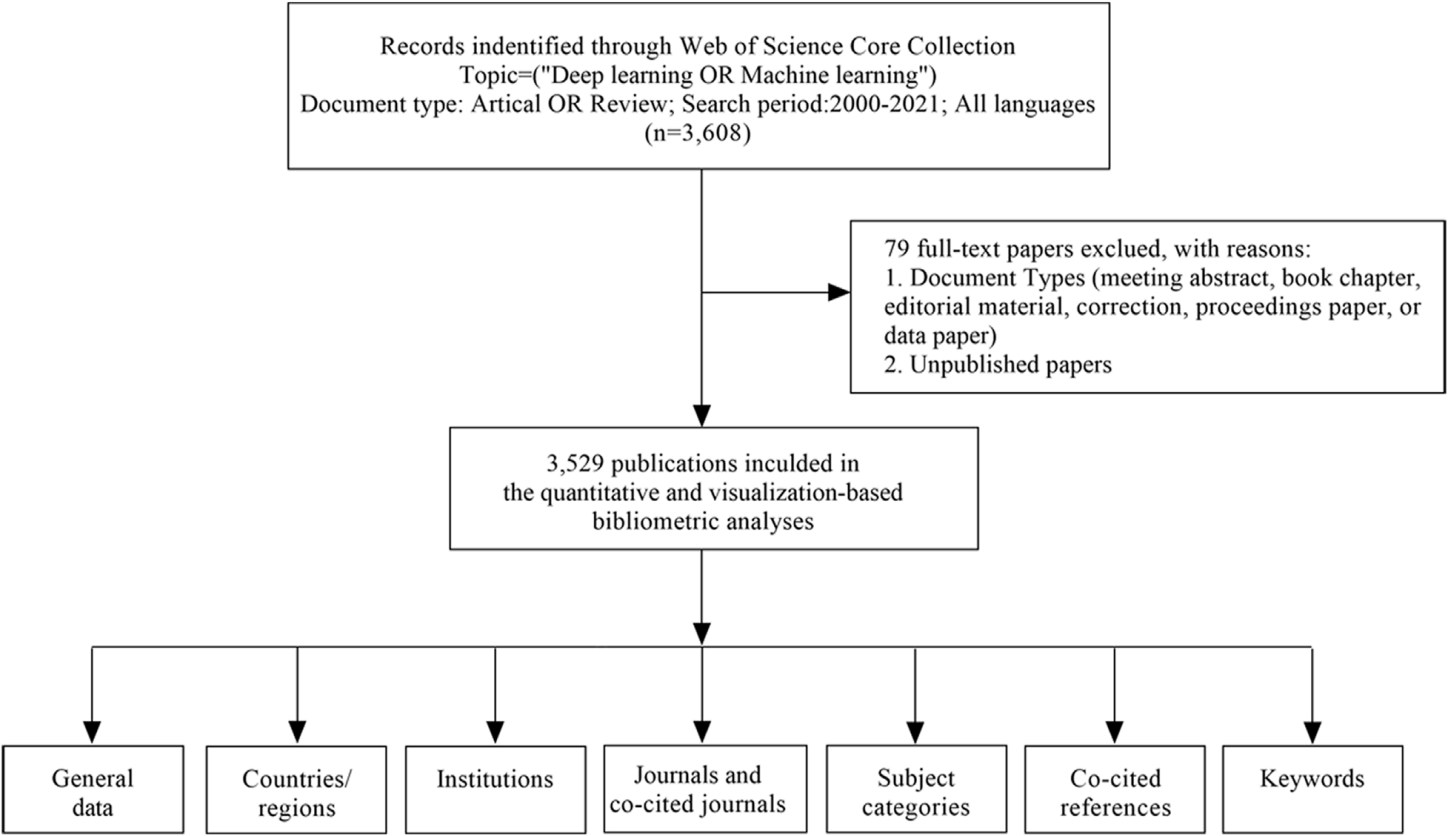
A frame flow diagram. The diagram showed details selection criteria for ABAM publications from WoSCC database and the steps of bibliometric analysis.

On 6 January 2022, we used the Web of Science Core Collection (WoSCC) to download data (2000–2021), which were independently verified by DX and ZZ. The following search terms were used: (“deep learning” OR “machine learning” OR “convolutional neural network*” OR CNN* OR RNN OR “Recurrent neural network*” OR “Fully Convolutional Network*” OR FCN*). The Web of Science category was “Biotechnology Applied Microbiology”, and documents were gathered. From studies, the following basic information was gathered: authors, abstract, title, institution, journal, keywords, country/region, and references. Studies indexed in the database were included, whereas the following were excluded: 1) book chapters, data papers, meeting abstracts and proceedings papers, repeated articles, and editorials, and 2) unpublished studies with limited data for analysis. In total, 79 duplicates were excluded. A study overview (search process and analyses) is provided (Figure 1).

We described publication traits, including country, institute, journals, and keywords. The H-index is an important indicator and was used to reflect the value of scientific research (Eyre-Walker and Stoletzki, 2013). The Literature Metrology websites; http://bibliometric.com/, VOSviewer (Leiden University, Leiden, Netherlands), and CiteSpace V (Drexel University, Philadelphia, PA, United States) were used to visualize collaborative networks in institutes/countries/keywords/journals and co-occurrence analyses. In CiteSpace, we conducted reference co-citation analyses, constructed knowledge maps, and identified burst keywords to generate new recurrent keywords (Chen, 2006).

## Results

### Article distribution by publication year

**FIGURE 2:**
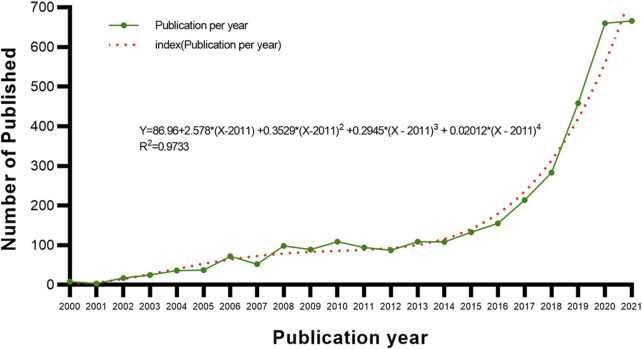
Trends in the number of publications on ABAM from 2000 to 2021.

The literature retrieval showed that the research on AI in this topic began in 2000. From 2000 to 2021, 3,529 papers were published, and AI with Biotechnology and Applied Microbiology (ABAM) related publication trends identified (Figure 2). Studies in this area are increasing year on year, and suggest the establishment of an important research trend.

### Institutes, countries, and regions

**FIGURE 3:**
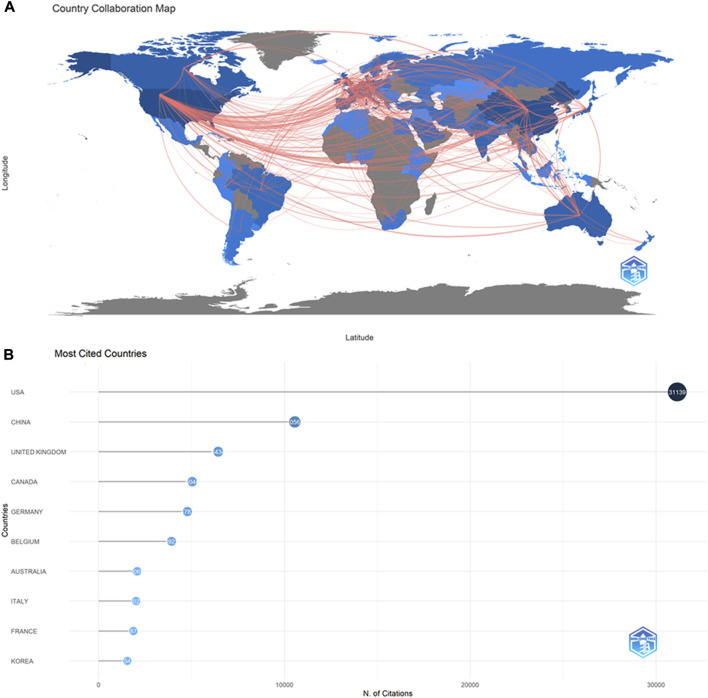
The cooperation of countries/regions contributed to publications. **(A)** Country Collaboration map. **(B)**Most Cited Countries.

We observed that 128 countries/regions published ABAM studies: collaborations between countries (Figure 3) and the top 10 countries (Table 1) are outlined. The United States published the most studies (1308), then China (826), Germany (258), and the United Kingdom (223). Some countries, such as United States, China, Germany, and United Kingdom, showed high centrality (marked by dark blue), indicating that these countries likely played an important role in research of this topic and made great contributions.

**TABLE 1:** Top 10 countries/regions and relevant institutions.

Rank	Countries/regions	Count	Total citations	H-index	Institutions	Count	H-index
1	United States	1308	31139	91	CHINESE ACAD SCI	85	26
2	China	826	10561	61	STANFORD UNIV	52	32
3	Germany	258	4783	44	SHANGHAI JIAO TONG UNIV	46	17
4	United Kingdom	223	6434	49	UNIV CAMBRIDGE	37	16
5	Canada	158	5040	37	CARNEGIE MELLON UNIV	36	16
6	Australia	128	2068	27	TSINGHUA UNIV	32	18
7	Italy	121	2022	28	UNIV ELECT SCI & TECHNOL CHINA	31	16
8	Japan	121	1327	30	HARVARD UNIV	31	30
9	South Korea	120	1547	25	TIANJIN UNIV	30	17
10	France	99	1875	29	UNIV WASHINGTON	30	17

**FIGURE 4:**
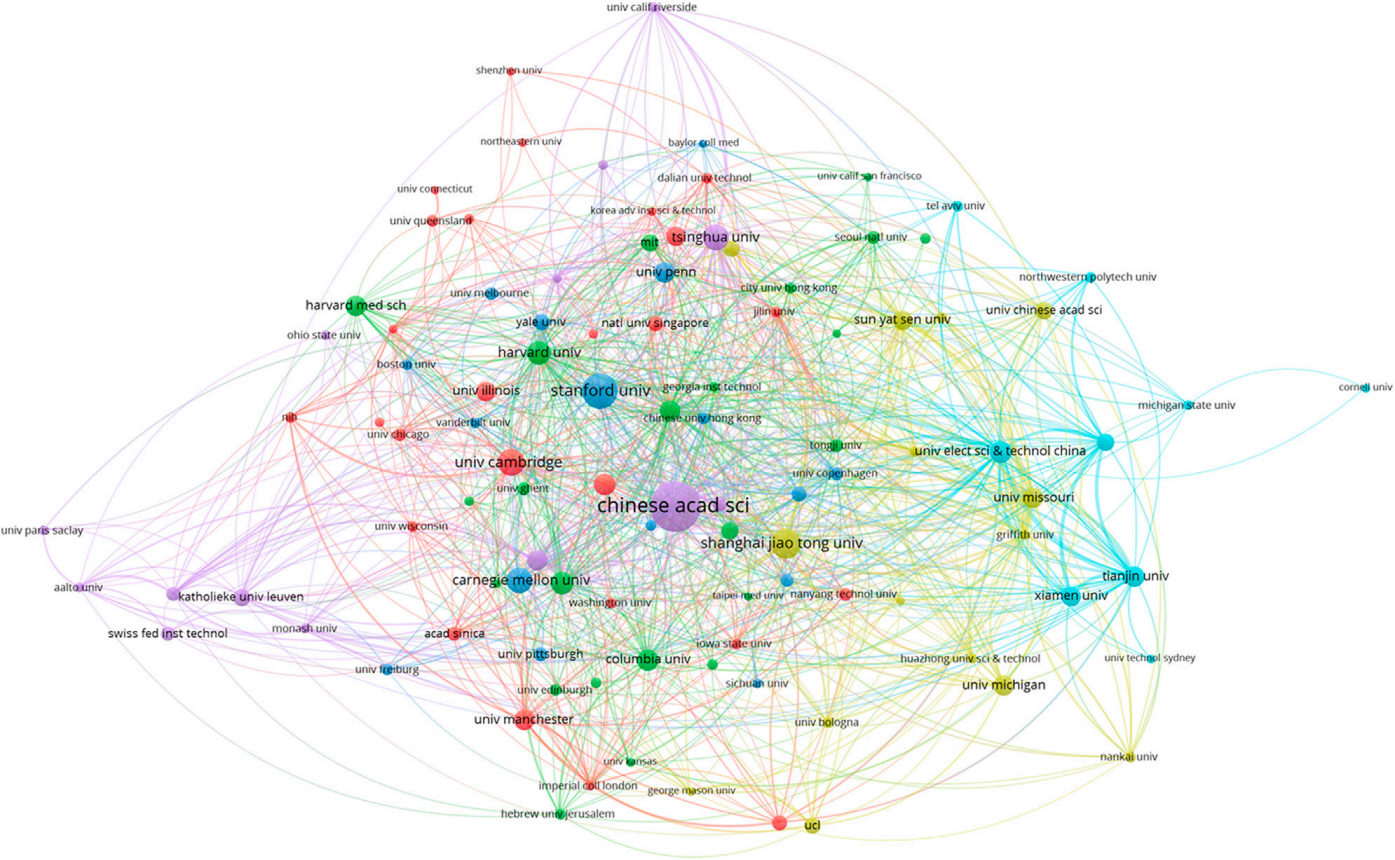
The cooperation of institutions contributed to publications.

We identified 584 institutes which contributed to ABAM publications; the top 10 are outlined (Table 1). Institutional collaborations are shown (Figure 4). The Chinese Academy of Sciences recorded the most publications (85), followed by the universities of Stanford (52), Shanghai Jiao Tong (46), and Cambridge (37).

Figure 4 emphasizes the close and complex cooperative relationship between different organizations. The VOSviewer platform can be used to analyze the centrality of organizations. The purple circle represents centrality, and the area of the circle is proportional to the centrality. The Chinese Academy of Sciences and Stanford University are the most prominent organizations, showing that they conducted more research in this area.

### Journals

**FIGURE 5:**
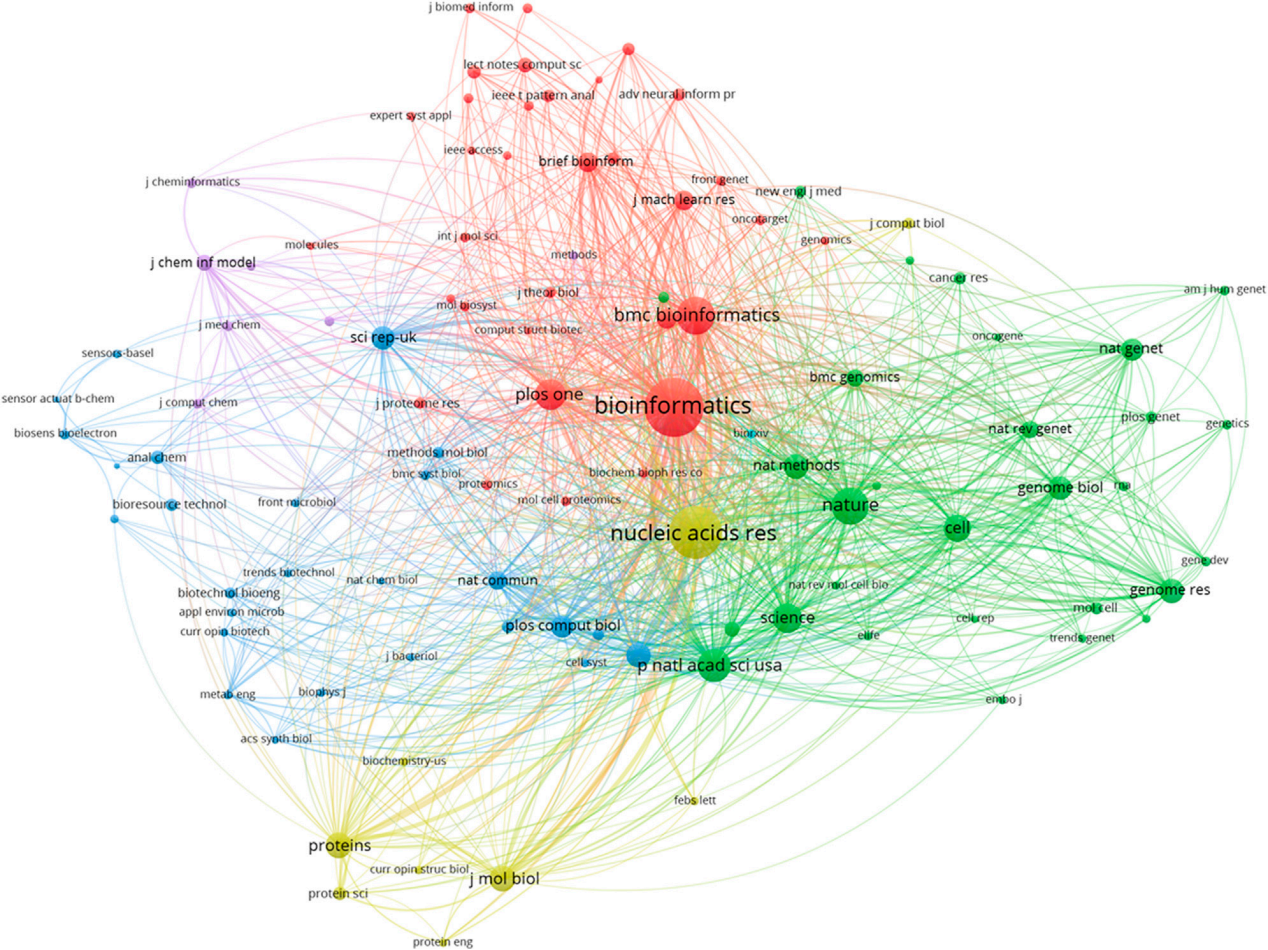
The network map of cited journals contributed to publications.

In any research field, referential relationships between academic journals often reflect knowledge exchange, where citing studies are knowledge frontiers, and referenced studies the knowledge basis. The top 10 references from studies (2000–2021) (Table 2) and collaborations between related journals (Figure 5) are outlined (Hall et al., 2009; Chang and Lin, 2011; Pedregosa et al., 2011; The ENCODE Project Consortium, 2012; Kingma and Ba, 2014; Srivastava et al., 2014; Alipanahi et al., 2015; LeCun et al., 2015; Zhou and Troyanskaya, 2015; Krizhevsky et al., 2017). Figure 5 shows that such journals as Bioinformatics, BMC Bioinformatics, Nature, Nucleic Acids Research, and PLoS One have higher centrality, and are the most popular journals for publishing research on this topic. The cooperative relationship between these journals is relatively balanced. This suggests that the research on the topic has aroused the interest of mainstream medicine and biology journals.

**TABLE 2:** Top 10 cited references on artificial intelligence for biotechnology and applied microbiology.

Rank	Source titles	Title of reference	Count	Interpretation of findings
1	NATURE	Deep learning.	203	This paper studied the back propagation algorithm of deep learning
2	J MACH LEARN RES	Dropout: a simple way to prevent neural networks from overfitting.	176	This study improved neural network performance in supervised learning tasks
3	NAT BIOTECHNOL	Predicting the sequence specificities of DNA-and RNA-binding proteins by deep learning.	151	This study used deep learning techniques to identify sequence specificities in DNA and RNA binding proteins
4	INT C LEARNING R	Adam: A method for stochastic optimization.	132	In this paper, a stochastic gradient descent optimization algorithm, based on the first derivative, was proposed for the first time. It was used for large data, sparse data processing, and super parameter easy adjustment.
5	J MACH LEARN RES	Scikit-learn: Machine learning, in python.	126	This article introduced scikit learning, a python module that integrated different contemporary machine learning algorithms
6	NAT METHODS	Predicting effects of noncoding variants with deep learning-based sequence model.	118	Based on deep learning, this study developed an algorithmic framework to identify functional effects from noncoding mutations
7	ACM T INTEL SYST TEC	LIBSVM: A library for support vector machines.	91	This study helped users apply support vector machine (SVM) to their applications.
8	COMMUN ACM	ImageNet classification with deep convolutional neural networks.	90	In this study, a large-scale deep CNN was used to classify 1.2 million high-resolution images
9	NATURE	An integrated encyclopedia of DNA elements in the human genome.	81	This study systematically mapped chromatin structure, transcription, transcription factor association, and histone modification regions.
10	SIGKDD EXPLORATIONS	The WEKA data mining software: an update.	79	The widely used, open source machine learning software Weka was introduced in this paper and allowed researchers access the latest technologies in machine learning.

**FIGURE 6:**
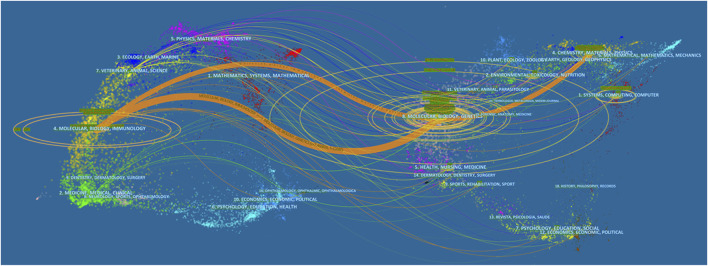
The dual-map overlay of journals contributed to publications.

A dual-map overlay of journals (Figure 6) was used to show citing and cited journals on the left and right, respectively, while citation relationships were reflected by colored paths—these analyses showed that studies published in Genetics/Molecular/Biology journals were typically published in Biology/Molecular/Immunology journals.

### References

Reference analysis is a vital bibliometric indicator; frequently cited studies typically and significantly influence their respective research fields. Using this approach, co-cited document-based clustering analyses generated connecting nodes and subfields for ABAM analyses.

**FIGURE 7:**
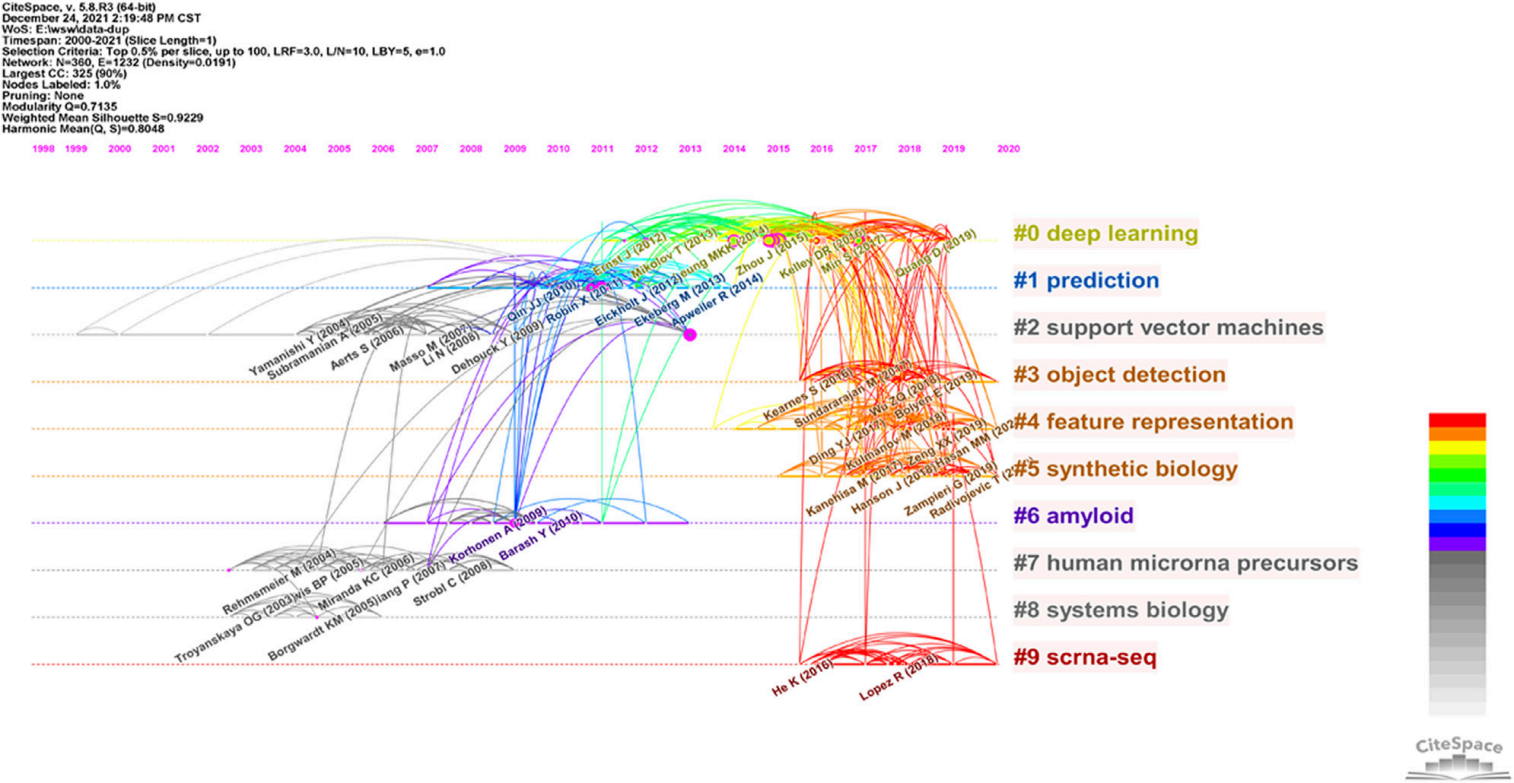
Reference co-citation map of publications on ABAM from 2000 to 2021.

We generated a co-citation reference network to measure the scientific relevance of related studies (Figure 7). Cluster setting parameters: top N% = 0.5 and # years per slice = 1. The Modularity Q score = 0.7135, which was > 0.5 and showed the network was reasonably separated into loosely coupled clusters. Weighted mean silhouette score = 0.9229, which was > 0.5, therefore cluster homogeneity was acceptable. Index items, as cluster markers, were extracted from studies. The largest cluster #0 was “deep learning” (Alipanahi et al., 2015), cluster #1 “prediction” (Kourou et al., 2015), cluster #2 “support vector machines (SVM)” (Furey et al., 2000), cluster #3 “object detection” (Hung et al., 2020), cluster #4 “feature representation” (Manavalan et al., 2019), cluster #5 “synthetic biology” (Wu et al., 2016), cluster #6 “amyloid” (Charoenkwan et al., 2021), cluster #7 “human microRNA precursors” (Wang et al., 2011), cluster #8 “systems biology” (Zou et al., 2015b), and cluster #9 “scRNA-Seq (single cell RNA-Sequencing)” (Arisdakessian et al., 2019).

### Keywords

Analysis of keywords can provide a summary of the topics of each study and explore the hotpots and directions in this research area.

**FIGURE 8:**
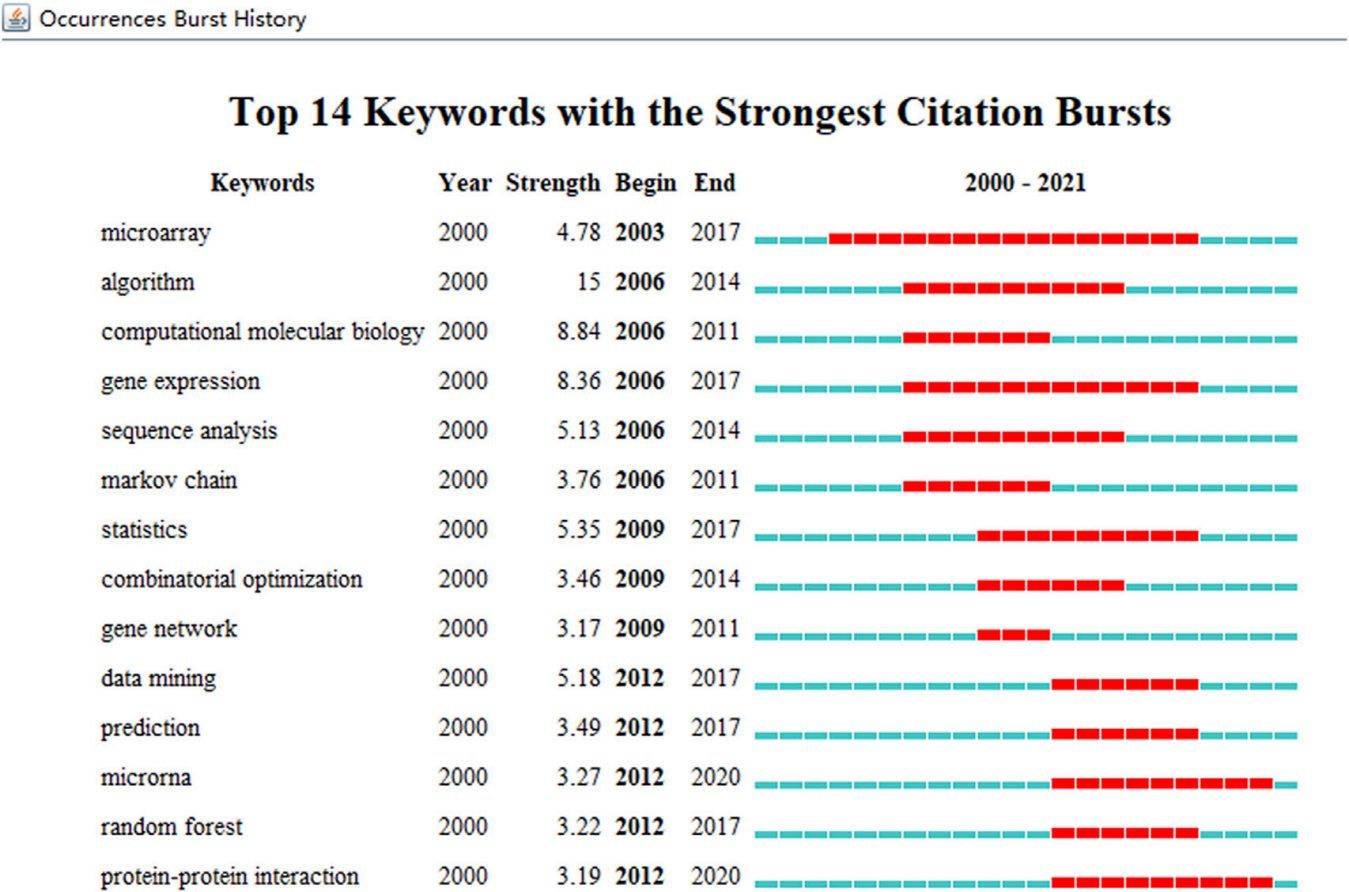
The keywords with the strongest citation bursts of publications on ABAM from 2000 to 2021.

Keywords extracted from ABAM studies were processed, and the top 20 are given in Table 3. Temporal hotspot trend shifts, on the basis of the top 14 keywords with the strongest citation bursts, were analyzed and included the following. The burst keywords in 2006–2011 were computational molecular biology (2006–2011), Markov chain (2006–2011), and gene network (2009–2011). The burst keywords in 2006–2014 were algorithm (2006–2014), sequence analysis (2006–2014), and combinatorial optimization (2009–2014). The burst keywords in 2003–2017 were microarray (2003–2017), gene expression (2006–2017), statistics (2009–2017), data mining (2012–2017), prediction (2012–2017), and random forest (2012–2017). The current research hotspots are microRNA (2012–2020) and protein–protein interaction (2012–2020) (Figure 8).

**TABLE 3:** Highly link strength of the top 20 occurrence keywords.

Rank	Keyword	Occurrence	Total link strength	Rank	Keyword	Occurrence	Total link strength
1	Machine learning	782	420	11	Prediction	44	52
2	Deep learning	318	183	12	Biomarkers	39	49
3	Classification	82	95	13	Algorithms	36	40
4	Convolutional neural network	72	51	14	Bioinformatics	33	51
5	Artificial intelligence	60	61	15	Cancer	30	44
6	Random forest	57	58	16	Genomics	28	39
7	Support vector machine	56	41	17	Clustering	25	20
8	Feature selection	52	62	18	Data mining	25	29
9	Gene expression	50	61	19	Rna-seq	21	28
10	Neural networks	46	41	20	Natural language processing	19	16

## Discussion

### General data

In this study, 3,529 ABAM papers, confirming to search terms and inclusion/exclusion criteria, were published between 2000 and 2021. The United States published most studies (1308, 26.6%), with China second (826, 16.8%). China had five of the top 10 institutions, with four in the United States, and one in the United Kingdom. The journal in which most publications were published was Bioinformatics, which majorly contributed to ABAM research. Additionally, the top 10 cited studies were examined: the top study was cited 203 times and was published by LeCun et al. in NATURE (LeCun et al., 2015). The second rated study was cited 176 times and published by Srivastava et al. in J MACH LEARN RES (Srivastava et al., 2014).

### Knowledge base

From previous studies, the application of deep learning related technologies to microbiology and biotechnology has been significant and generated many research achievements. As indicated (Figure 6), when we clustered co-cited references, key clustering nodes identified knowledge bases in this research field: #0 “deep learning”, #1 “prediction”, #2 “SVM”, #3 “object detection”, #4 “feature representation”, #5 “synthetic biology”, #6 “amyloid”, #7 “human microRNA precursors”, #8 “systems biology”, and #9 “scRNA-Seq”. Herein, we describe the knowledge bases according to different clusters.

In #0 “deep learning”, a DeepBind software tool, based on deep learning, was developed by Alipanahi et al. (2015), and identified DNA and RNA binding protein sequence specificity. The tool was used to develop regulatory process models in biological systems and identify pathogenic variants. In other work, Beck et al. (2020) generated a deep learning-based, pre-trained, drug-target interaction model, Molecule Transformer-Drug Target Interaction, which identified commercially available drugs targeting SARS-CoV-2 proteins.

In #1 “prediction”, Tsubaki et al. (2019) studied end-to-end representation learning of compounds and proteins, and developed a Compound-Protein Interactions (CPI) prediction strategy for virtual screening in drug discovery by combining protein convolution neural networks (CNN) and compound graph neural networks. In other work, Almagro Armenteros et al. (2017) developed a prediction algorithm based on deep neural networks which relied only on sequence information for protein subcellular localization.

In #2 “SVM”, Ozer et al. (2020) showed that SVM provided solutions for high-throughput data analyses and contextualization; the approach rapidly determined timelines for invasive cancer diagnostics and treatment, and provided solutions for biomedical, bioengineering, and clinical applications. In other work, a SVM technology model constructed by Zhang et al. (2018) used joint information from multiple bone turnover markers, which improved diagnostic efficiency for osteoporosis, almost in perfect agreement with the dual-energy X-ray absorptiometry.

In #3 “object detection”, the approach by Zhang et al. (2020), exploited a deep object detection technique and was used to study contacts between protein secondary structure elements, and predict tertiary structural protein topology. Einhäuser et al. (2017) developed a foveal object detector to detect eye movement, which significantly reduced metabolic costs and computational complexity, and provided insights on visual system evolution with eye movement.

In #4 “feature representation”, an effective feature representation learning model ACPred-FL was developed by Wei et al. (2018), and used to rapidly and accurately identify new Anti-cancer peptides (ACPs)in many candidate proteins and peptides. The learning method developed by Peng et al. (2020) was based on feature representation learning and deep neural network (DTI-CNN), and was used to predict drug-target interactions and reduce time and experimental costs. In other research, from deep representation learning features with 107 dimensions, Lv et al. (2020) devised a sub-Golgi protein localization identification method, which exploited one feature type to accurately predict sub-Golgi protein localization.

#5 “synthetic biology” is a logical extension of recombinant technology or genetic engineering fields (Katz et al., 2018). Using integrated synthetic biology, Nguyen et al. (2021) developed a wearable face-mask, with a lyophilized CRISPR sensor, to non-invasively detect SARS-CoV-2 at room temperature within 90 min. Cubillos-Ruiz et al. (2021) proposed that synthetic biology could be used to program living cells with therapeutic functions; their cell-based therapeutic design is currently undergoing rapid development in medicine, and may provide effective treatment solutions for human diseases.

In #6 “amyloid”, Charoenkwan et al. (2021) generated the first scorecard-based predictor for the accurate analysis, prediction, characterization, and identification of amyloid, on a large scale, to generate functional information for therapeutic intervention strategies. Cerebral amyloid-β (Aβ) is an Alzheimer’s disease (AD) trait. Machine learning methods were used to identify cognitive performance and demographic variables for noninvasive testing of Aβ deposition, which can detect the effect of anti-amyloid drugs in the non-dementia population (Ko et al., 2019).

In #7 “human microRNA precursors”, Zheng et al. (2020) used CNN and RNN approaches to automatically extract complex RNA sequence features to efficiently detect and predict human pre microRNAs. Kamenetzky et al. (2016) identified a novel pre-microRNA in the *Echinococcus multilocularis* genome using a machine learning approach, which could help control and prevent the global zoonotic infectious disease alveolar echinococcosis.

In #8 “systems biology”, Reel et al. (2021) integrated different machine learning prediction algorithms to analyze different omics data to identify new biomarkers for systems biology. In their research, Weiskittel et al. (2021) outlined how systems biology algorithms layer machine learning and biological components could provide system-level analyses of single-cell omics data to clarify complex biological mechanisms. The powerful combination of systems biology, single cell omics, and machine learning could promote further, beneficial biomedical research.

In #9 “scRNA-Seq”, in an unbiased manner in single cells, scRNA-Seq assesses functions in individual cells and cell-to-cell variability (Lin et al., 2020). Based on deep neural networks, Arisdakessian et al. (2019) formulated an interpolation algorithm Deepimpute based on DNN. Dropout layers and loss function were used to learn data patterns and to deal with gaps in scRNA-Seq data. He et al. (2020) developed DISC, a deep learning imputation model with semi-supervised learning for single cell transcriptomes. DISC can deduce gene expression and structures obscured by dropouts, enhanced gene and cell structures, recovered poor gene expression, and improved cell identification. Using machine learning methods (deep learning) combined with scRNA-Seq datasets, issues such as reducing dimensions, missing values, denoizing sc data, and explaining zero expansion, can be solved. Machine learning methods can be exploited to comprehensively process scRNA-Seq data, improve follow-up analyses in stem cells, identify cell subsets, and support regenerative medicine and cell therapy strategies (Yan et al., 2021).

### Research frontiers and hotspots

Typically, keywords are used to concentrate on contemporary research concepts, while burst keywords represent research frontiers and emerging trends. CiteSpace was used to capture burst keywords, from which two research frontiers were identified: microRNA (2012–2020) and Protein-Protein Interaction (PPIs) (2012–2020). Importantly, we hypothesize these keywords exemplify future research frontiers.

MicroRNAs are noncoding single stranded RNAs that regulate development and gene transcription. Predicting and identifying connections between miRNAs and disease using AI-related methods is highly significant for unraveling pathogenic, preventative, prognostic, and pathological mechanisms implicated in diseases.

Zou et al. (2015a) predicted correlations between microRNAs and disease using two approaches: KATZ combined social network analysis and machine learning, while CATAPULT was a supervised machine learning method. Both were applied to 242 known associations between microRNAs and disease, and used 3-fold cross validation and leave-one-out cross-validation to evaluate method performance.

Wen et al. (2018) used the deep learning-based approach DeepMir Tar and extracted 750 features from a relatively large data set at different levels to predict human miRNA target sites. DeepMir Tar provided a new way to reveal miRNA biological function, as well as gene therapy and drug discovery for human diseases.

In large-scale RNA sequencing studies, Liu *et al.* developed a computational model called MirTarget which predicted genome-wide miRNA targets. Machine learning methods were used to train miRNA targeting feature data with miRNA binding and target down-regulation features, thus MirTarget showed better performances when compared with other algorithms (Liu and Wang, 2019).

Zheng et al. (2020) used CNN and RNN models to predict human pre-miRNAs; sequences were combined with predicted pre-miRNA secondary structures as input features to avoid feature extraction and selection processes by hand. Models were easily trained for handling training datasets; they demonstrated low generalization errors and were satisfactory for test datasets (Zheng et al., 2020).

Protein–protein interactions are very important in such cell life activities as transcriptional regulation, signal transduction, and drug signal transduction. Study of PPIs has become a research hotspot in bioinformatics. However, it is time-consuming and costly to identify PPIs using experimental methods (Chen et al., 2019).

People are more inclined to use artificial intelligence methods, like machine-learning, to automatically identify PPIs, which helps understanding of the molecular roots of disease on one hand, and provides new ideas for drug research and development on the other hand. Also, this effectively reduces experimental costs (Yu et al., 2021).

Based on a deep learning algorithm, Sun et al. (2017) designed a stacked autoencoder and investigated sequence-based PPIs predictions; the prediction accuracy of different external datasets was 87.99%–99.21%. These high-throughput methods increased our understanding of protein roles, disease etiology, and therapy design.

Hashemifar et al. (2018) developed a Direct Physical Protein-Protein Interactions (DPPI) deep learning framework, which modeled and predicted PPIs from sequence information. By adopting a deep, Siamese-like CNN which used high-quality experimental PPI data, evolutionary information from a predicted protein pair, and combined these data with random projection and data enhancement, PPIs were successfully predicted (Hashemifar et al., 2018).

Zeng et al. (2019) formulated DeepPPISP, a novel end-to-end deep learning framework. To examine local contextual features, authors used a sliding window to acquire neighbor features from target amino acids. To analyze global sequence features, a text CNN extracted features from protein sequences. To predict PPI sites, local contextual and global sequence characteristics were combined (Zeng et al., 2019).

Sequence-based deep learning technologies have been successfully used to predict PPIs. However, Yang et al. (2020) indicted these methods only focus on sequence information and ignore structural information in PPI networks. Such information, including degree, location, and adjacent nodes in graphs, are vital for PPI predictions. Theses authors generated a graph-based deep learning method for predicting PPIs, and demonstrated an accuracy of 99.15%, which improved on existing sequence-based methods (Yang et al., 2020).

In their method based on deep learning, Liu-Wei et al. (2021) developed deepviral, which predicted PPIs between humans and viruses. The method processed protein sequences and phenotypic characteristics to reveal infectious disease mechanisms and elucidate potential treatment methods (Liu-Wei et al., 2021).

## Conclusion

We generated an objective, systematic, and comprehensive bibliometric analysis of scientific studies associated with deep learning, machine learning, CNN, RNN, and FCNs in ABAM. Moreover, we identified the research basis, future trends, and current hotspots in this field. Identified knowledge bases were: deep learning, prediction, SVMs, object detection, feature representation, synthetic biology, amyloid, human microRNA precursors, systems biology, and scRNA-Seq. Furthermore, microRNAs and PPIs were identified as future research frontiers and trends.

We identified some study limitations; publications over an extended period (2000–2021) were gathered, therefore, some studies were incomplete and may have introduced publication bias into our research, potentially affecting analysis outcomes.

## Abbreviations

AI, Artificial Intelligence; WoSCC, Web of Science Core Collection; CPI, Compound-Protein Interactions; ACPred-FL, Anti-Cancer Peptide Predictor with Feature Representation Learning; ACPs, Anti-cancer peptides; CRISPR, Clustered Regularly Interspaced Short Palindromic Repeats; SARS-CoV-2, Severe Acute Respiratory Syndrome Coronavirus 2; AD, Alzheimer’s Disease; CNN, Convolutional Neural Network; Aβ, amyloid-β; RNN, Recurrent Neural Network; RNA, Ribonucleic Acid; DNN, Deep Neural Networks; DISC, Deep learning Imputation model with semi-supervised learning for Single Cell transcriptomes; PPI, Protein-Protein Interaction; DPPI, Direct physical Protein-Protein Interactions; SVM, Support Vector Machine.
